# Comparative transcriptomes of adenocarcinomas and squamous cell carcinomas reveal molecular similarities that span classical anatomic boundaries

**DOI:** 10.1371/journal.pgen.1006938

**Published:** 2017-08-07

**Authors:** Eric W. Lin, Tatiana A. Karakasheva, Dong-Jin Lee, Ju-Seog Lee, Qi Long, Adam J. Bass, Kwok K. Wong, Anil K. Rustgi

**Affiliations:** 1 Department of Medicine, Division of Gastroenterology, University of Pennsylvania, Philadelphia, Pennsylvania, United States of America; 2 Abramson Cancer Center, University of Pennsylvania, Philadelphia, Pennsylvania, United States of America; 3 Department of Systems Biology, Division of Cancer Medicine, UT MDACC, Houston, Texas, United States of America; 4 Department of Biostatistics, Epidemiology and Bioinformatics, University of Pennsylvania, Philadelphia, Pennsylvania, United States of America; 5 Department of Medical Oncology, Dana Farber Cancer Institute, Boston, Massachusetts, United States of America; 6 Department of Medicine, Division of Hematology and Medical Oncology, New York University Langone Medical Center, New York, New York, United States of America; 7 Department of Genetics, University of Pennsylvania, Philadelphia, Pennsylvania, United States of America; Baylor College of Medicine, UNITED STATES

## Abstract

Advances in genomics in recent years have provided key insights into defining cancer subtypes “within-a-tissue”—that is, respecting traditional anatomically driven divisions of medicine. However, there remains a dearth of data regarding molecular profiles that are shared across tissues, an understanding of which could lead to the development of highly versatile, broadly applicable therapies. Using data acquired from The Cancer Genome Atlas (TCGA), we performed a transcriptomics-centered analysis on 1494 patient samples, comparing the two major histological subtypes of solid tumors (adenocarcinomas and squamous cell carcinomas) across organs, with a focus on tissues in which both subtypes arise: esophagus, lung, and uterine cervix. Via principal component and hierarchical clustering analysis, we discovered that histology-driven differences accounted for a greater degree of inherent molecular variation in the tumors than did tissue of origin. We then analyzed differential gene expression, DNA methylation, and non-coding RNA expression between adenocarcinomas and squamous cell carcinomas and found 1733 genes, 346 CpG sites, and 42 microRNAs in common between organ sites, indicating specific adenocarcinoma-associated and squamous cell carcinoma-associated molecular patterns that were conserved across tissues. We then identified specific pathways that may be critical to the development of adenocarcinomas and squamous cell carcinomas, including Liver X receptor activation, which was upregulated in adenocarcinomas but downregulated in squamous cell carcinomas, possibly indicating important differences in cancer cell metabolism between these two histological subtypes of cancer. In addition, we highlighted genes that may be common drivers of adenocarcinomas specifically, such as *IGF2BP1*, which suggests a possible link between embryonic development and tumor subtype. Altogether, we demonstrate the need to consider biological similarities that transcend anatomical boundaries to inform the development of novel therapeutic strategies. All data sets from our analysis are available as a resource for further investigation.

## Author summary

In clinical practice, the organ in which a cancer arises typically classifies it. However, developments in our understanding of cancer have revealed that this method overlooks key aspects of cancer biology relevant to both disease prevention and treatment. In fact, work characterizing the genetic make-up of cancers arising in a single organ has revealed that a shared organ of origin does not necessarily imply biological similarity (i.e. not all lung cancers share similar biological and molecular properties). While this approach, known as “within-a-tissue subtyping,” identifies key differences between cancers that arise in a single organ, a broader perspective may highlight important biological similarities between cancers across organs. Here we utilize this second approach, or “across-tissue subtyping,” to gain insight into similarities between cancers (of different organs) that share the same histology—or appear similarly under a microscope. Using publicly available data from The Cancer Genome Atlas (TCGA), we compare gene expression of two major classes of solid tumors—adenocarcinomas (which arise from cells that form glands) and squamous cell carcinomas (which arise from flattened cells that form physical barriers). We identify several genes and biological pathways that may be common to adenocarcinomas and serve as targets for highly versatile therapies.

## Introduction

The classification of cancers for specific and tailored clinical management has been a topic of ongoing study. Historically, cancers have been classified primarily by the organs in which they originate—a convenient strategy that aligns with traditional anatomical divisions of medicine. Accordingly, many currently available treatments, such as surgery and radiation, are anatomically driven, and tumors arising in different sites are often managed in different ways (and by different subspecialty divisions), even if they possess histological similarity (i.e. squamous cell carcinomas arising in the head and neck, as opposed to the anogenital region). Meanwhile, systemic treatments such as chemotherapy largely do not discriminate between histologically distinct tumors arising within the same organ (i.e. squamous cell carcinomas and adenocarcinomas of the esophagus). These tumors, on the whole, are commonly treated in the same manner. In fact, current guidelines suggest similar treatment of squamous cell carcinomas (SCCs) and adenocarcinomas (ADCs) in the esophagus as well as the cervix, despite observations of differences in prognosis, risk factors, patterns of recurrence, and even response to treatment [1–3].

Recent efforts to re-classify cancers for both clinical and research purposes have addressed this latter issue by utilizing “within-a-tissue” subtyping, identifying molecular signatures that can distinguish between tumors arising within the same organ [4–7]. This has facilitated the development of biologically relevant targeted therapies for particular cancer subtypes, such as molecular subtypes of lung cancer. However, a less common approach has been “across-tissues” analysis [8], which focuses on more global molecular patterns that may transcend traditional anatomic boundaries. This latter approach may provide key insights into common cancer-associated genes and pathways that span several organ sites, which may facilitate drug development by allowing for the generation of therapeutics that can impact a wider population. For example, categorizing cancers based on mutational burden and immunogenicity has been critical to the broad application of immunotherapies, such as immune checkpoint inhibitors, across a wide array of solid malignancies. In addition, recent perspectives have called for a unified approach to the management and study of SCCs, based on observed similarities in risk factors, genomics, and functional biology [9].

Expanding on this concept, we sought to characterize further the molecular and functional similarities between SCCs and ADCs. ADCs represent the most common type of cancer as a whole, comprising over two-thirds of all cases (of solid and hematologic malignancies combined) in the United States from 2009 to 2013 [10]. Typically originating in organs containing native glandular tissue, adenocarcinomas are the predominant subtype of cancers arising in the breast, colon, prostate, lung, pancreas, stomach, ovary, kidney, and, in the western hemisphere, the esophagus [11]. To our knowledge, there is no literature explicitly comparing adenocarcinomas across tissues of origin at the genomic and transcriptomic levels.

In this study, we utilized data generated by The Cancer Genome Atlas (TCGA) Research Network to study molecular and functional differences between SCCs and ADCs arising within the same organ site. By focusing on organs in which both these subtypes arise, namely the esophagus, lung and uterine cervix, we aimed to determine whether tissue of origin or another property, such as histology, contributes more significantly to observed variation in molecular signatures among tumors. Using a transcriptome-centered approach, we demonstrate here that differences in histology account for more variation than anatomic or tissue origin. Furthermore, via differential gene expression, DNA methylation, and microRNA analysis, we identify 1733 genes, 346 CpG sites, and 42 miRNAs that distinguish ADCs and SCCs between organs, and show that histology correlates with distinct transcriptional and epigenetic programs that are largely consistent across organ sites. Lastly, utilizing pathway and survival analysis, we highlight common markers of ADCs that not only have prognostic value but may also have functional roles in tumorigenesis, thus serving as potential targets for broad therapeutic intervention.

## Results

### Histology accounts for more transcriptome variation than tissue of origin

**Fig 1:**
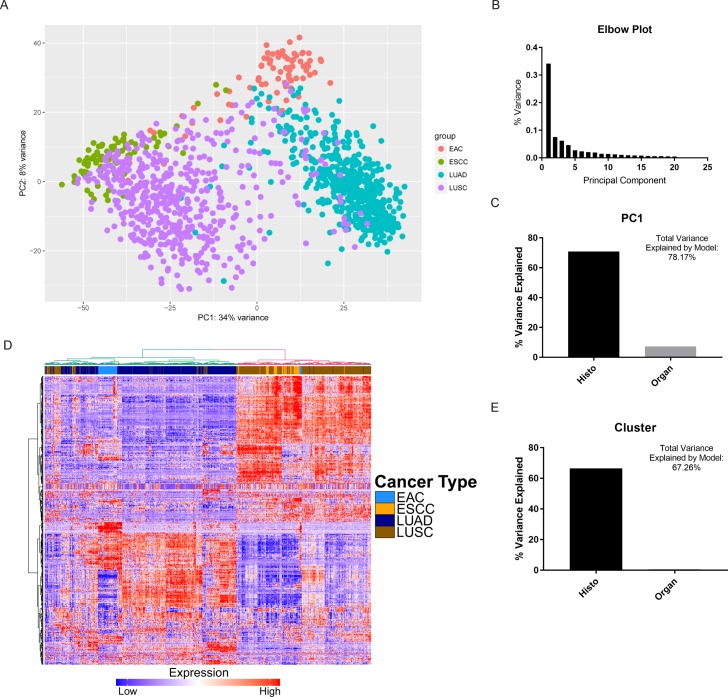
Histology explains a greater degree of gene expression variation than organ site. (A) Principal Component Analysis (PCA) plot depicting two largest components of variance in gene expression in four cancer types. (B) Elbow plot showing the proportion of variance explained by the first 20 principal components (PC1 explains 0.34 or 34% of total variance). (C) Bar chart depicting relative importance (percent variance explained) by histology and organ site, respectively, based on a multiple linear regression model with PC1 as the response variable. Regression model created was [PC1 = 47.2 × Histo + 21.66 × Organ − 42.85], where explanatory variables were demarcated as Histo (0 = SCC, 1 = ADC) and Organ (0 = Esophagus, 1 = Lung). (D) Heatmap of differentially expressed genes between esophageal cancers and lung cancers, with hierarchical clustering. (E) Bar chart depicting relative importance (percent variance explained) by histology and organ site, respectively, based on a multiple linear regression model with Cluster number (1 or 2) as the response variable. Regression model created was [Cluster = 0.80 × Histo + 0.12 × Organ + 1.01], where explanatory variables were demarcated as Histo (0 = ADC, 1 = SCC) and Organ (0 = Lung, 1 = Esophagus).

To identify the most significant drivers of variation in gene expression, we first performed a principal component analysis (PCA) of gene expression data from 80 esophageal adenocarcinomas (EAC), 81 esophageal squamous cell carcinomas (ESCC), 533 lung adenocarcinomas (LUAD), and 502 lung squamous cell carcinomas (LUSC) (Fig 1A). After ruling out significant batch effects (S1 Fig), we discovered that the greatest component driving gene expression variation (PC1) accounted for significantly more variation (34% of total) than other components (Fig 1B). Using multiple regression, we next determined the relative importance of histology and organ site to predicting PC1. We found that of the variance that our regression model could explain, histology accounted for 90.7%, while organ site only accounted for 9.3% (Fig 1C). We built on these findings using hierarchical clustering, which revealed two main clusters that appeared to be defined by histology: EAC tended to cluster with LUAD, while ESCC tended to cluster with LUSC (Fig 1D). Interestingly, while the majority of EAC samples formed its own subcluster, this pattern was less apparent in ESCC. When we determined the relative contribution of histology or organ site to cluster number via multiple regression, we noted a much stronger contribution from histology (Fig 1E), with histology accounting 98.9% of the variance explained by the regression model (organ site accounted for 1.1%). These findings suggest that histology may be of greater importance to a tumor’s molecular profile than its tissue of origin.

### Histology-driven gene expression patterns are consistent across organs

To understand how consistent histology-driven molecular patterns were across organs, we first compared mRNA expression between ADCs and SCCs in the esophagus and lung. We identified 3443 differentially expressed genes between EAC and ESCC, and 4198 differentially expressed genes between LUAD and LUSC, with 1733 genes that were common in both comparisons (S2 Fig and S1 File). Among these were known markers of squamous cell-fate determination, *TP63* and *SOX2*, which have a role in both the development of normal squamous tissue in the esophagus as well as tumorigenesis along the squamous lineage in the lung [12,13] and esophagus [14,15]. Other notable genes relatively upregulated in SCCs were keratins *KRT14* and *KRT17*; *DSC3*, a member of the desmocollin family (a component of intercellular desmosomes); *FAT2*, an atypical cadherin found to be induced by ΔNp63α (isoform of TP63) and promote invasion in LUSC [16]; *EGFR*, a key growth factor receptor in many cancers; *CCNA1*, which encodes for cyclin A1, a cell cycle regulator; and *WNT3A*, a member of the Wnt family of signaling proteins, which also may have a role in SCCs [17]. Upregulated in ADCs were glandular markers such as mucins *MUC3A* and *MUC13*; claudins (which modulate tight junction permeability) *CLDN2* and *CLDN18*, which has recently been targeted therapeutically in advanced gastric and gastroesophageal junction adenocarcinomas [18]; drivers of cell differentiation such as *GATA6*; hepatic nuclear factors such as *HNF1B*, *FOXA2*, *FOXA3*; and *SPINK1* (serine protease inhibitor Kazal-type 1), which has been associated with a number of gastrointestinal and genitourinary cancers [19].

**Fig 2:**
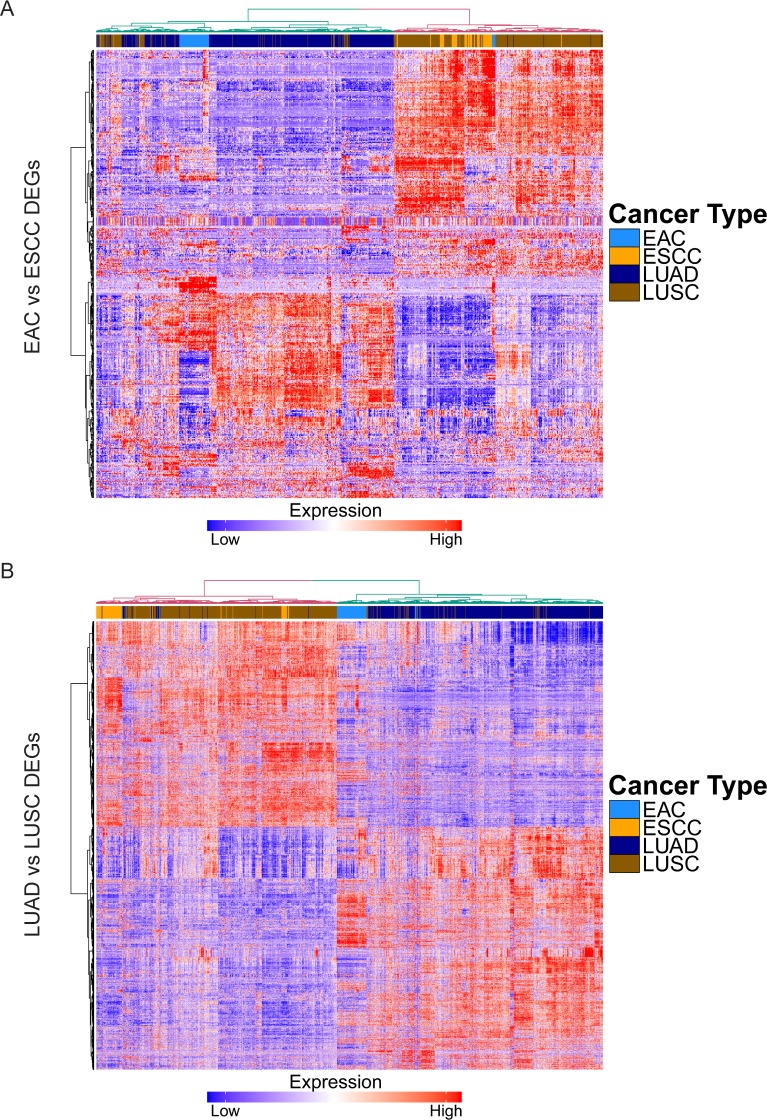
Global molecular patterns defined by histology are consistent across both esophagus and lung. (A) Heatmap depicting mRNA expression of DEGs between EAC and ESCC in ADCs and SCCs of esophagus and lung, with hierarchical clustering. (B) Heatmap depicting mRNA expression of DEGs between LUAD and LUSC in ADCs and SCCs of esophagus and lung, with hierarchical clustering.

Next, we evaluated if global patterns of histology-driven gene expression were similar across organ sites by constructing heatmaps with clustering using the differentially expressed genes (DEGs) identified in the esophagus and lung, respectively (Fig 2A and 2B). We found that histology drove a clear and consistent pattern of expression in both the esophagus and lung. Importantly, these DEGs were able to distinguish ADCs from SCCs across both organs after applying hierarchical clustering. These data indicate gene expression profiles determined by histology are largely consistent across different organ sites.

### Histology-driven epigenetic patterns are similar across organs

**Fig 3:**
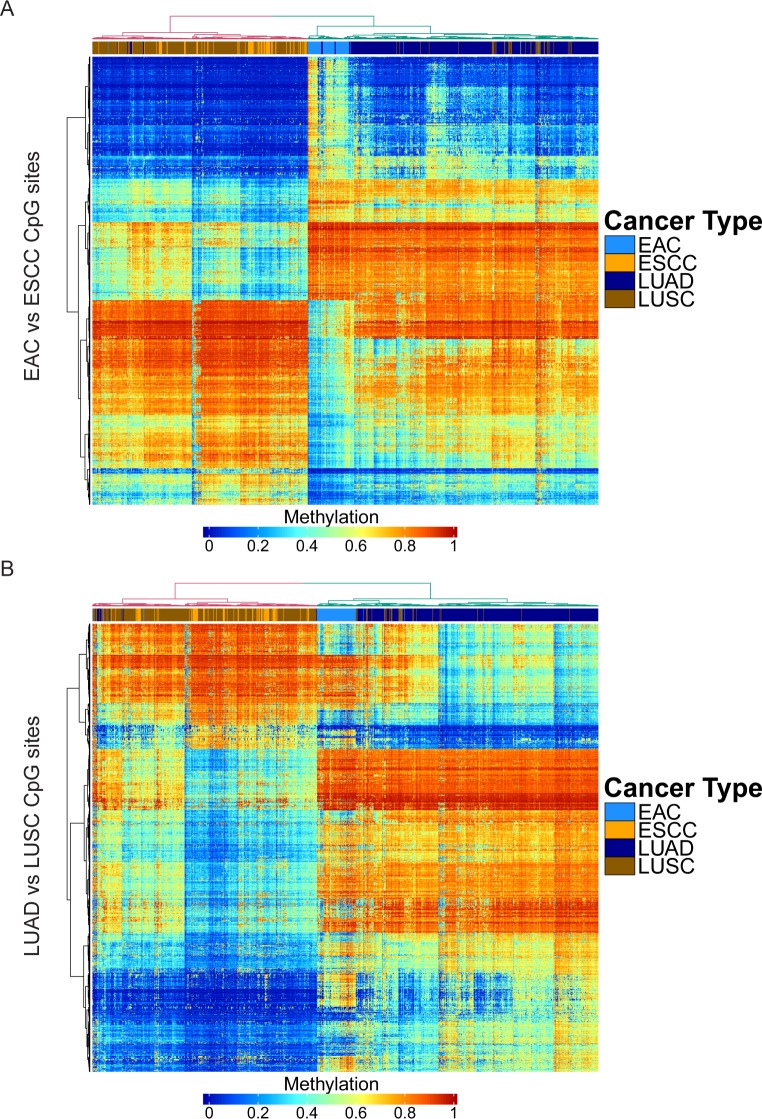
Overall DNA methylation patterns defined by histology are consistent across esophagus and lung. (A) Heatmap depicting DNA methylation of differentially methylated CpG sites between EAC and ESCC in ADCs and SCCs of esophagus and lung, with hierarchical clustering. (B) Heatmap depicting DNA methylation of differentially methylated CpG sites between LUAD and LUSC in ADCs and SCCs of esophagus and lung, with hierarchical clustering.

To determine if the patterns observed in differential gene expression in ADCs versus SCCs were associated with epigenetic changes, we compared DNA methylation in each histological subtype. We identified 1734 differentially methylated CpG sites between EAC and ESCC, 1650 differentially methylated CpG sites between LUAD and LUSC, with 346 CpG sites in common between the comparisons (S2B Fig and S2 File). When we observed patterns of DNA methylation and applied hierarchical clustering, we again found that the cancers grouped by histology and not by organ site (Fig 3A and 3B). Interestingly, while EAC and LUAD appeared to form distinct subclusters within the ADC cluster, ESCC and LUSC were more homogeneous in DNA methylation profile and thus did not form separate subclusters.

We then sought to identify particularly important genes by intersecting differentially expressed genes with those that were differentially methylated. We identified 174 such genes in the esophagus, 193 genes in the lung, and 33 common genes between them. Genes that were downregulated and hypermethylated in ADCs were squamous markers such as *DSC3* and *KRT5*, as well as transcription factors such as *FOXE1* and *TP73*, whose isoforms have demonstrated both tumor suppressor and oncogenic properties [20]. Interestingly, *TP73* overexpression in SCCs coincided with *TP63* overexpression in SCCs relative to ADCs (S2C Fig). The predominant isoform of *TP63* in squamous epithelia and SCCs is ΔNp63 [21], which has been demonstrated previously to reversibly inhibit *TP73*-dependent transcription by direct promoter binding or physical interaction with the p73 protein, leading to decreased apoptosis [22]. We did not, however, detect a consistent downregulation of p73 target genes [23], including those involved in apoptosis (*PMAIP1* and *BBC3*), which may be due to selective inhibition of specific downstream targets or activation by alternative regulators (S2C Fig). By contrast, genes that were upregulated and hypomethylated included mucins like *MUC1*; *SERPINA1*, a serine protease inhibitor that acts in the liver and lung; *HNF1B*, a transcription factor that regulates in renal and pancreatic development; and *ME3*, a mitochondrial malic enzyme whose deletion was shown recently to confer lethality in *SMAD4*-deleted pancreatic adenocarcinoma [24]. These data demonstrate that histological subtype-specific patterns of gene expression correspond to changes in epigenetic profiles.

### Histology-defined microRNA expression

**Fig 4:**
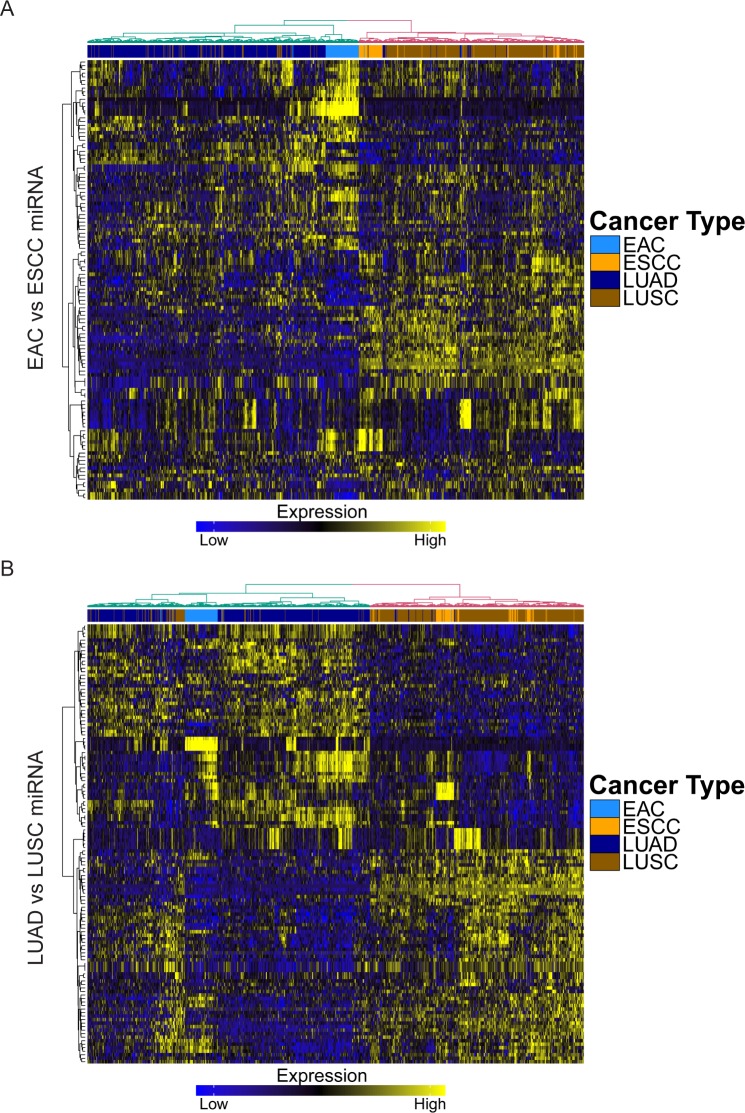
Global miRNA expression patterns defined by histology are consistent across both esophagus and lung. (A) Heatmap depicting miRNA expression of differentially expressed miRNAs between EAC and ESCC in ADCs and SCCs of esophagus and lung, with hierarchical clustering. (B) Heatmap depicting miRNA expression of differentially expressed miRNAs between LUAD and LUSC in ADCs and SCCs of esophagus and lung, with hierarchical clustering.

We then analyzed other potential influences on gene expression. MiRNAs have gained significant attention due to their post-transcriptional regulation of gene expression by destabilizing mRNA and silencing translation. Using miRNA expression data acquired from TCGA, we compared the levels of specific miRNAs in ADCs and SCCs of the esophagus and lung. We identified 118 differentially expressed miRNAs in the esophageal comparison, and 125 in the lung, with 42 overlapping miRNAs (S2D Fig and S3 File). The miRNAs relatively overexpressed in SCCs included miR-994, which has been found to be part of intronic sequence of ΔNp63 and is highly expressed keratinocytes [25], as well as miR-224, which has been implicated in the progression of colorectal cancer and non-small cell lung cancer (NSCLC) [26,27]. On the contrary, miRNAs upregulated in ADCs included miR-215, which has been shown to modulate gastric tumor cell proliferation by targeting *RB1* [28], and miR-375, which has been observed to be upregulated in lung adenocarcinoma but downregulated in lung squamous cell carcinoma, and promotes cell proliferation by decreasing levels of *ITPKB*, a putative tumor suppressor [29,30]. Interestingly, also downregulated in ADCs was miR-149, which was demonstrated previously to have tumor suppressor capacity in breast cancer migration and invasion by targeting small GTPases Rap1a and Rap1b [31]. Furthermore, similar to mRNA expression, patterns of miRNA expression were largely conserved for ADC and SCC across both esophagus and lung (Fig 4A and 4B). These findings suggest that similar patterns of miRNA expression also govern differences in histology, which can be observed across different primary organ sites.

### Distinct functional pathways are upregulated in ADC versus SCC

**Fig 5:**
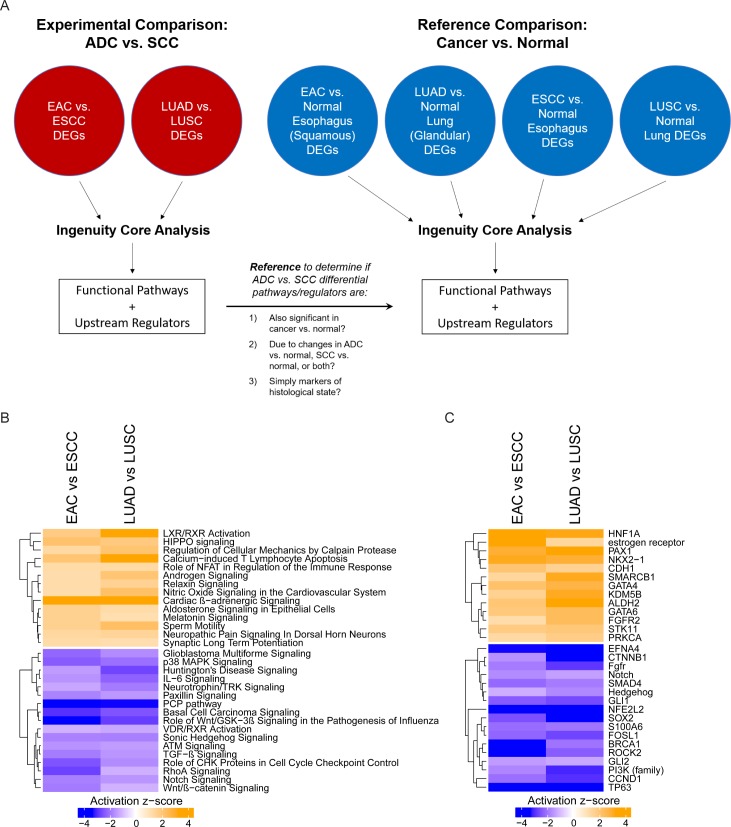
Pathway and upstream regulator analysis using DEGs between ADC and SCC. (A) Schematic illustrating workflow from differential gene expression analysis to pathway and upstream regulator analysis, along with use of reference comparisons (cancer vs. normal). (B) Putative functional pathways that may play a role in the development of ADC and SCC, by activation z-score. (C) Potential upstream regulators with a functional impact on ADC versus SCC, by activation z-score.

Given the histologically-driven patterns observed in transcriptome, epigenome and post-transcriptional profile, we next performed pathway analysis based on differentially expressed genes to identify functional pathways specific to ADCs or SCCs. Using DEGs from our previous ADC vs. SCC comparisons (i.e. EAC vs. ESCC), we performed pathway analysis with Ingenuity software. As a reference, we also performed pathway analysis using DEGs from cancer vs. normal comparisons (i.e. EAC vs. normal esophagus) (Fig 5A). This reference comparison allowed us to determine whether differential pathways identified in ADC vs. SCC were significantly altered in cancer biology (relative to normal), and whether these differences were due to changes in ADC, SCC, or both. Importantly, the LUAD vs. normal lung (both glandular) and ESCC vs. normal esophagus (both squamous) comparisons provided insight into whether differential pathways were simply markers of a specific histological state, since pure histological markers would be unlikely to result in differences observed in these comparisons.

In our analysis, there were several pathways relatively upregulated in ADCs compared to SCCs (Fig 5B). Intriguingly, the only pathway that was also consistently upregulated in ADC versus normal tissue was liver X receptor / retinoid X receptor (LXR/RXR) activation (S3A Fig). LXR/RXR activation was also downregulated in SCCs relative to normal tissue (S3A Fig), suggesting a dichotomous function in these different histological subtypes. Furthermore, differences in LXR/RXR activation were maintained in the LUAD vs. normal lung and ESCC vs. normal esophagus comparisons, indicating that it is likely more than a simple marker for glandular histology (S3A Fig). Other pathways relatively upregulated in ADCs included HIPPO signaling, Relaxin signaling, and Androgen Signaling; however, these pathways were mostly downregulated in all cancers relative to normal tissue (S3A Fig). The differentially expressed genes corresponding to each pathway are listed in a supplementary table (S1 Table).

There were several pathways that were relatively downregulated in ADCs compared to SCCs. Some of these pathways were also downregulated in ADCs relative to normal tissue, including p38 MAPK Signaling and Paxillin Signaling. The remaining pathways were upregulated in SCCs relative to both ADCs and normal tissue, suggesting an important functional role in the carcinogenesis of SCCs. This included Basal Cell Carcinoma Signaling, the planar cell polarity (PCP) pathway, and Wnt signaling. Of note, Basal Cell Carcinoma Signaling is closely related to Sonic Hedgehog (SHH) Signaling, which has been associated with the carcinogenesis of SCCs of the oral mucosa, uterine cervix, esophagus and lung [32–35], where it may have a role in mediating stemness via transcription factors like *SOX2* [34,35]. Furthermore, aberrant Wnt signaling and non-canonical Wnt/PCP signaling, which normally regulates cell shape via the cytoskeleton [36], have been hypothesized to have a unique role in SCCs [17], and may represent a functional distinction between SCCs and ADCs [37].

Expanding on this pathway analysis, we investigated potential upstream regulators driving changes in gene expression (Fig 5B). Notably upregulated in ADCs relative to SCCs was *STK11* (or LKB1), loss of which has been previously shown to induce adeno-to-squamous differentiation of lung tumors in mice [38]; *GATA4* and *GATA6*, important for cell differentiation and commonly amplified in EAC and gastric cancers [4]; and *NKX2-1*, a commonly used marker for lung adenocarcinomas. Furthermore, the estrogen receptor was found to be relatively upregulated in ADCs versus SCCs and normal tissue. Several genes were also found to be likely upstream regulators of SCC formation. For instance, *CDH1*, which codes for E-cadherin, a membrane protein important for cellular adhesion, was relatively downregulated in SCCs, which aligns with reports of its methylated and suppressed expression in oral SCCs [39]. Other notable genes relatively upregulated in SCC were *GLI1*, a downstream mediator of hedgehog signaling; *SMAD4*, which is involved in TGF-β signaling and is commonly is deleted in EAC and LUAD [4,5]; *CCND1*, a cyclin that is commonly amplified in ESCC [4]; and *TWIST1*, thought to be important in epithelial-to-mesenchymal transition [40]. The activation of these upstream regulators in each cancer type relative to corresponding normal tissue is shown in a supporting figure (S3B Fig).

### Identifying genes important for survival in ADC

**Fig 6:**
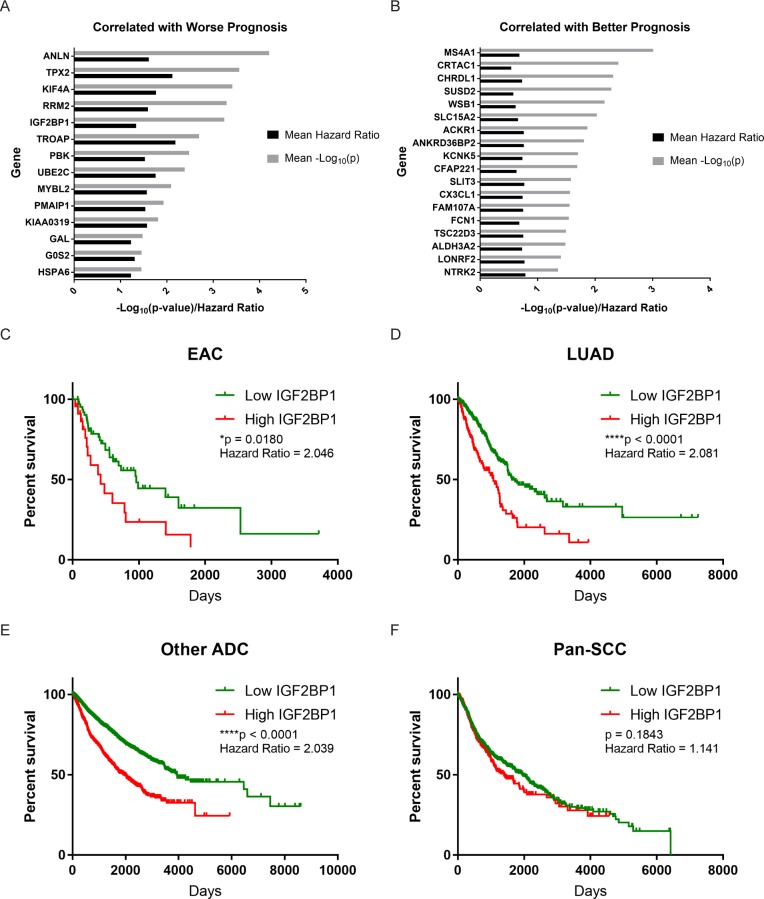
Genes correlated with survival in ADCs. (A) Genes whose expression is correlated with worse survival in ADCs. (B) Genes whose expression is correlated with better survival in ADCs. (C) Kaplan-Meier curve depicting survival in high *IGF2BP1* and low *IGF2BP1* expression groups in EAC (log-rank test). (D) Kaplan-Meier curve depicting survival in high *IGF2BP1* and low *IGF2BP1* expression groups in LUAD (log-rank test). (E) Kaplan-Meier curve depicting survival in high *IGF2BP1* and low *IGF2BP1* expression groups in pooled ADCs, excluding EAC and LUAD (log-rank test). (F) Kaplan-Meier curve depicting survival in high *IGF2BP1* and low *IGF2BP1* expression groups in pooled SCCs (including ESCC and LUSC) (log-rank test).

We next sought to highlight additional gene candidates that may serve as prognostic markers among ADCs, given the lack of unifying features previously described in the literature. Using the 500 genes showing the greatest variance in expression, we performed Cox proportional hazards survival analysis to find genes significantly correlated with survival outcomes in both EAC and LUAD. We discovered 109 such genes in EAC, and 691 genes in LUAD, with 32 genes in common between them (Fig 6A and 6B). Genes whose increased expression was correlated with worse survival outcomes included *TPX2*, *KIF4A*, *IGF2BP1*, and *HSPA6*. On the contrary, genes whose expression was correlated with a better prognosis included *MS4A1* (CD20), *SUSD2*, and *CX3CL1* (Fractalkine). As an example, we constructed Kaplan-Meier curves for *IGF2BP1*, or IMP-1, which we described previously as a modulator of tumor growth in colorectal cancer [41]. Not only did higher expression of *IGF2BP1* correlate with poorer outcomes in EAC and LUAD (Fig 6C and 6D), but the same trend was seen when we pooled several other ADCs from the TCGA Pan-Cancer dataset (including cancers of the breast, prostate, endocervix, endometrium, ovary, pancreas, stomach, kidney, colon, rectum, and thyroid) (Fig 6E). Notably, the same trend was not observed in a pooled SCC dataset (including cancers of head and neck, esophagus, lung, and ectocervix) (Fig 6F). Intriguingly, while ESCC and LUSC had 88 and 206 genes associated with survival, respectively, they shared no genes that had the same directional association with outcomes.

### Validation: Expression patterns in the uterine cervix

**Fig 7:**
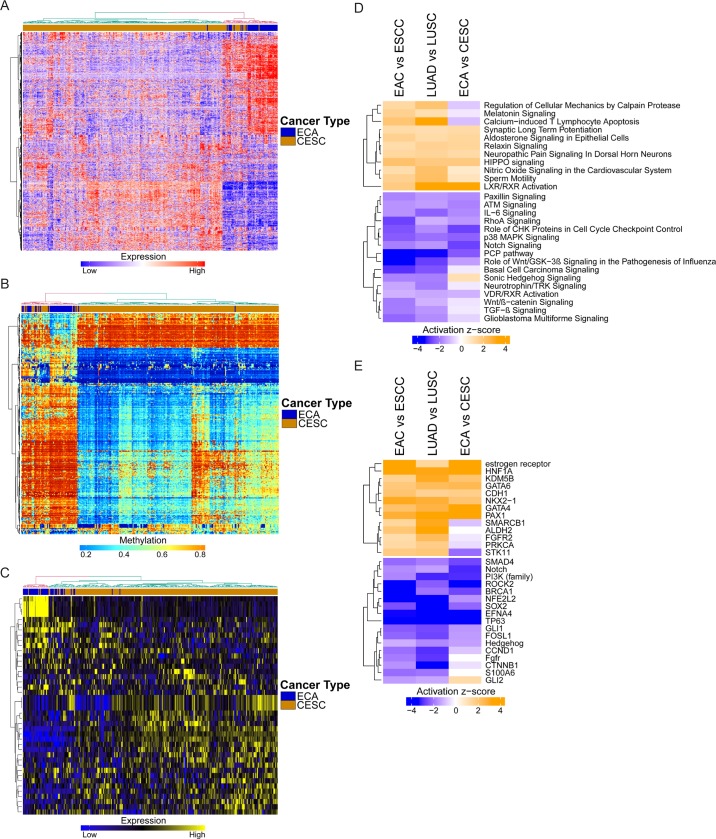
Validation in endocervical adenocarcinoma and cervical squamous cell carcinoma. (A) Heatmap depicting mRNA expression of common DEGs between ADC and SCC (esophagus and lung) in the cervix, with hierarchical clustering. (B) Heatmap depicting DNA methylation of common differentially methylated CpG sites between ADC and SCC (esophagus and lung) in the cervix, with hierarchical clustering. (C) Heatmap depicting miRNA expression of common differentially expressed miRNAs between ADC and SCC (esophagus and lung) in the cervix, with hierarchical clustering. (D) Pathways identified using DEGs from ECA versus CESC, compared to results from the esophagus and lung. (E) Upstream regulators identified using DEGs from ECA versus CESC, compared to results from the esophagus and lung.

As a validation, we attempted to reproduce our results in a third organ site in which both ADCs and SCCs arise: the uterine cervix. While the predominant subtype of cancer in the cervix is squamous cell carcinoma (CESC) associated with human papilloma virus (HPV) infection, several types of adenocarcinoma, specifically endocervical, endometroid, and mucinous adenocarcinoma, can also develop. Using data acquired from TCGA, we pooled 44 samples of these various types of cervical ADCs (ECA) and compared them with 254 CESC samples by using gene expression, DNA methylation, and miRNA expression data acquired from the esophagus and lung comparison. Upon clustering, we found that the same genes, methylated CpG sites, and miRNAs distinguishing histology in the esophagus and lung were also largely able to distinguish histology in the cervix (Fig 7A–7C).

The pathways we had identified previously to be important to ADC and SCC, respectively, also maintained similar patterns in the cervix, with deviations only in Regulation of Cellular Mechanics by Calpain Protease, Melatonin Signaling, and Calcium-induced T Lymphocyte Apoptosis in pathways favoring ADCs (Fig 7D). LXR/RXR Activation was once again upregulated in ECA relative to CESC. In pathways that were relatively downregulated in ADCs, Notch Signaling and the PCP pathway maintained strong associations with SCC; however, there was a weaker relationship in the cervix between squamous histology and Basal Cell Carcinoma Signaling, Sonic Hedgehog Signaling, or TGF-β Signaling. We also performed upstream regulator analysis of ECA and CESC, again observing similar patterns to the histological subtypes of the esophagus and lung (Fig 7E). Notable differences included *ALDH2*, *STK11* in genes relatively upregulated in ADCs, and *CTNNB1* and *GLI2* in genes relatively downregulated in ADCs. Altogether, histology-driven differences in transcriptome, epigenome, as well as pathways and upstream regulators, were largely consistent across all three organ sites studied.

## Discussion

Our analyses have revealed major differences in the transcriptomic and epigenomic profile of ADCs and SCCs, but major similarities among ADCs and SCCs spanning several anatomic sites. Importantly, histology was the greater determinant of molecular variation than organ site, which further emphasizes the need to re-classify tumors and re-structure current treatment paradigms. While genomic subtyping studies have demonstrated the need to consider molecular characteristics (and biological relevance) when managing tumors arising within the same organ, we also highlight the premise that some molecular features may be shared by tumors arising in different organs. Capitalizing on this property of cancer could lead to the development of widely applicable targeted therapies.

Importantly, we highlighted several specific properties that may unify ADCs, including a large collection of genes, CpG methylation sites, and miRNAs. Specifically, we identified LXR/RXR activation as having a putative role in the development of ADC. Indeed, several recent reports have highlighted liver X receptors as potential therapeutic targets, as they appear to regulate aerobic glycolysis (the Warburg effect) and lipogenesis [42], which malignant cells depend upon to sustain rapid proliferation, while also modulating antitumor immunity [43]. In fact, one study utilized an inverse agonist SR9243 to inhibit LXR activity, suppressing tumor growth in several colon, prostate, lung and pancreatic adenocarcinoma cell lines [42]. Curiously, our analyses also indicated that LXR/RXR activation was downregulated in SCCs relative to normal tissue. This is in line with a previous report that described the spontaneous development of peripheral squamous cell lung cancer in mice with ablated LXRα,β (*NR1H3-/-* and *NR1H2-/-*) [44]. Taken together, this evidence points to a possible dichotomous role of LXRs in ADCs versus SCCs and may indicate key differences in cell metabolism between these histological subtypes of solid malignancies. Despite these findings, however, other studies on LXRs have reported a downstream antiproliferative effect that can be harnessed by using LXR agonists (oxysterols) in several cancer types, including multiple ADCs [45]. It is clear, therefore, that further investigation on the precise role of LXRs in different contexts is required.

In addition, we identified candidate genes that correlate with patient outcomes and may have a functional role in ADC carcinogenesis, such as *IGF2BP1*. Intriguingly, increased *IGF2BP1* expression was found to be associated with worse survival in EAC, LUAD, and a pooled dataset of other ADCs spanning breast to colon (Fig 6C–6F). The same pattern was not observed in a pooled SCC dataset. *IGF2BP1* is an mRNA binding oncofetal protein, not normally expressed in adult tissues, that we previously demonstrated to have a role in modulating colorectal tumor growth *in vitro* and *in vivo*, and may also play a role in early metastasis [41]. Similar findings were reported previously in ovarian and breast adenocarcinomas [46,47]. This association further emphasizes the importance of developmental pathways in tumorigenesis, and specifically indicates a possible link between embryonic drivers of differentiation and histological subtypes in cancer. Notably, there have been efforts to develop inhibitors of *IGF2BP1*, with some recent success in the identification of a compound that selectively kills *IGF2BP1* expressing cells [48].

Liver X receptors and *IGF2BP1* are just two examples of therapeutic targets that may be particularly effective in ADCs. Our analysis has revealed an entire catalog of gene candidates for further study, including those that correlate with survival outcomes, though an association with survival is not a prerequisite for being an effective target (i.e. estrogen receptor positivity in breast cancer). Notably, our findings also have implications for the use of currently available therapeutics in ADCs versus SCCs. For example, our upstream regulator analysis highlighted several putative regulators that have greater activity in SCCs than ADCs, such as GLI1 and the PI3K family. These results suggest that SCCs may have greater sensitivity to GLI1 or Hedgehog inhibitors, as well as PI3K inhibitors, than ADCs. Another finding with potential therapeutic relevance is *TP73* methylation and low expression in ADCs. *TP73* is a known tumor suppressor [20], and its methylation could contribute to reduced apoptosis in response to chemo- or radiotherapy relative to SCCs [3]. One could hypothesize that the use of DNA methylation inhibitors, such as 5-azacitidine or decitabine, could restore sensitivity to cytotoxic therapy in ADCs, although this requires experimental validation.

We then noted that although global histology-driven patterns were conserved in esophagus and lung, some of these similarities may have been due to a common embryonic precursor, as both the esophagus and lung are derived from the foregut endoderm [49]. To address this, we tested our findings in a third organ, the uterine cervix, which develops from the paramesonephric ducts and is mesodermal in origin. We found that histology-driven differences were largely maintained even in the cervix (Fig 7A–7C). There were some notable exceptions, however, which could be related to different embryonic tissue origin or differences in other factors such as the local tumor microenvironment. For example, *STK11* was discovered to be relatively downregulated in ESCC and LUSC, but relatively upregulated in CESC (Fig 7E). These findings are consistent with other reports: while loss of *STK11* has been associated with lung, skin, and head and neck SCCs [50], inherited loss of *STK11*, as in Peutz-Jeghers syndrome, has been associated with endometrial adenocarcinoma and a rare variant of endocervical carcinoma, minimal deviation adenocarcinoma of the endocervix [51]. Differences observed between the cervix and the esophagus and lung could also be due to distinct risk exposures. For instance, *ALDH2*, which is important for acetaldehyde (a metabolite of ethanol and an ingredient in tobacco smoke) metabolism and whose deficiency increases the risk for esophageal and head and neck SCCs, was found to be relatively downregulated in ESCC and LUSC, but was not significantly different when comparing ECA and CESC (Fig 7E). This could be due to direct exposure of the aerodigestive tract and respiratory mucosa to acetaldehyde via alcohol consumption and cigarette smoking, respectively, in *ALDH2-/-* individuals.

Lastly, there were some technical limitations to our *in silico* analysis. First, TCGA datasets can be susceptible to batch effects, although when we assessed the data, variation due to batch was determined to be of relatively insignificant impact (S1 Fig). Similarly, although TCGA datasets are generally large, they may not be representative of the general population, and critically, samples sent for sequencing are often not purely tumor cells and may contain associated stromal and immune components, as well as normal tissue. Additionally, because we were primarily interested in global molecular patterns, our analysis was not sensitive to relatively rare variants such as those described in genomic and mutational analyses. It should be noted that highlighted candidate pathways and genes need to be verified further for functional significance with additional *in vitro* or *in vivo* analyses.

## Conclusion

These analyses further emphasize the need to re-categorize tumors in a biologically relevant manner—not only as a practice in pathology and research, but also in the clinic. Over the last decade, molecular subtyping efforts have already called for a shift in approach to viewing cancers—such that esophageal carcinoma, for instance, would not be viewed as a single entity [4–6], but as multiple diseases with distinct characteristics and patterns of behavior. While our findings distinguishing between histological subtypes support this premise, we also propose that broader strategies of classification to direct research efforts and clinical management may be more aligned to the pragmatic concerns of drug development while still being biologically relevant. Consider the example of immune checkpoint inhibitors, which are being widely applied across cancers.

In this report, we emphasize the call for a unified approach to the study, prevention, and treatment of SCCs, and on possible commonalities underlying ADCs. Despite apparent heterogeneity among ADCs, we have identified patterns of gene expression, functional pathways, and prognostic markers that may similarly unify our approach to ADCs. Furthermore, our analyses have provided a catalog of additional candidate genes and markers that can be further investigated. To our knowledge, this is the first study identifying common features among adenocarcinomas arising from different organ sites.

## Methods

### Data acquisition

Publicly available gene expression, DNA methylation, and microRNA (miRNA) data from TCGA were downloaded from the Genomic Data Commons (GDC) Data Portal and pre-processed via the *TCGAbiolinks* R package [52]. Details regarding specific data type and subsequent analysis are described below. Survival outcomes data were acquired from the University of California Santa Cruz (UCSC) Xena at http://xena.ucsc.edu.

### Differential gene and microRNA expression

Harmonized gene expression data (*HTSeq* counts)—to allow for easy comparison between cancer types—from GDC was downloaded for esophageal carcinoma (TCGA-ESCA), lung adenocarcinoma (TCGA-LUAD), lung squamous cell carcinoma (TCGA-LUSC), and cervical and endocervical carcinoma (TCGA-CESC), and assembled into a matrix using *TCGAbiolinks* [52]. Potential batch effects were visualized by constructing a PCA plot by batch and cancer type, using the R package *DESeq2* [53]. Considering insignificant batch effects and to avoid masking biologically significant differences, no corrections for batch were applied. Using the *edgeR* package in R [54], the counts data were filtered for low counts (less than 1 count per million in greater than 50% of each group, i.e. esophageal adenocarcinoma vs. esophageal squamous cell carcinoma) and normalized within samples using the trimmed means of M-values (TMM) method [55]. Differentially expressed genes (DEGs) were filtered using the *glmTreat* function in *edgeR*, which tests for significant differences relative to a set log_2_-fold change (logFC) cutoff of 0.5. Results were then filtered by a false discovery rate (FDR) of 0.05.

Harmonized microRNA expression data for ESCA, LUAD, LUSC, and CESC were acquired from the GDC Data Portal and preprocessed via *TCGAbiolinks* [52]. Low counts were filtered as above differential expression analysis was performed using *edgeR*. A logFC cutoff of 0.5 and FDR cutoff of 0.05, as above, were used.

### DNA methylation analysis

Legacy DNA methylation data (Illumina Human Methylation 450, aligned to hg19) from GDC was downloaded via *TCGAbiolinks* [52]. Using the *TCGAanalyze_DMR* function, we searched for differentially methylated CpG sites using beta-values, with a cutoff difference in beta-values of 0.25 and adjusted p-value of 10^−20^. The results from these data were then integrated with the differential gene expression data.

### Principal component analysis, hierarchical clustering and visualization

To identify global patterns in gene expression, raw counts were first quantile-normalized within and across samples, using the *TCGAanalyze_Normalization* function, then transformed using the *varianceStabilizingTransformation* function in *DESeq2* to reduce heteroscedasticity (chosen over log transformation to minimize variance at low count values) [53]. Principal component analysis (PCA) was performed using the *prcomp* function in R. Hierarchical clustering was performed in R by Euclidean distance and the ward.D2 method. To determine the relative contribution of explanatory variables (Histology and Organ) to variance, we first constructed multiple linear regression models for response variables “PC1” and “Cluster”, respectively, using the *lm* function in R. We then utilized the *relaimpo* package [56], which refers to the Lindeman, Merenda, Gold (1980) method of *R*^*2*^ partitioning by average over orders [57], in order to determine the contribution to variance of each explanatory variable.

For visualization, the PCA were plotted by cancer type using the *plotPCA* function in *DESeq2* [53]. For heatmaps, normalized gene expression data (as described above) and miRNA expression data were further scaled to a mean of 0 and standard deviation of 1, while for DNA methylation, β-values were used directly in visualization. All heatmaps were constructed using the *ComplexHeatmap* R package [58]. Bar charts were constructed in GraphPad Prism.

### Pathway and upstream regulator analysis

Pathway analysis and identification of upstream regulators, using differentially expressed genes as input, were performed using QIAGEN Ingenuity Pathway Analysis software (using the core analysis feature). The same cutoffs as described in the differential gene expression analysis were used to filter significant genes. To determine significant pathways across different organs, we used the comparison analysis function in Ingenuity. Significant pathways were filtered by activation z-score (absolute value ≥ 1.0) and adjusted p-value (≤ 0.05), in the ADC vs. SCC experimental comparisons.

Specifically, the DEGs from seven comparisons were initially used as input for Ingenuity (Fig 5A). These included three ADC vs. SCC comparisons (esophagus, lung, and cervix), as well as four reference cancer vs. normal comparisons. As discussed in the text, these reference comparisons help to determine whether differential pathways identified in ADC vs. SCC were also significantly altered in cancer biology (relative to normal), and whether these differences were due to changes in ADC, SCC, or both. Additionally, the LUAD vs. normal lung (both glandular) and ESCC vs. normal esophagus (both squamous) comparisons provided insight into whether differential pathways were simply markers of a specific histological state, since changes in these comparisons would not be expected for simple histological markers.

### Survival analysis

Survival outcomes for TCGA samples were downloaded from UCSC Xena. To identify genes that have potential prognostic and functional significance, we only analyzed the top 500 genes with greatest variance in either ADCs or SCCs. Given the continuous nature of gene expression data, we used a cox proportional hazards model to identify genes correlated with survival. In order to improve sensitivity, no correction for multiple testing was applied. Survival was visualized by plotting Kaplan-Meier curves in GraphPad Prism 7.0. Importantly, TCGA pan-cancer data was acquired from UCSC Xena. Pooled ADC data contained 14 different ADC types with 4934 samples. Pooled SCC data contained 4 SCC types with 1346 samples. For survival, high expression and low expression groups were determined by cutoff at the 75^th^ percentile.

### Statistical analysis

Statistical analysis for principal component analysis, linear correlation, differential expression, and Cox proportional hazards were performed in R. Pathway statistical analysis was built-in to Ingenuity. Log-rank tests for Kaplan-Meier were performed using GraphPad Prism.

## Supporting information

S1 Fig Principal Component Analysis plot depicting batch effect versus group (cancer type) effect. (TIF)

S2 Fig ADC versus SCC of esophagus and lung show overlap in DEGs, DNA methylation and miRNA expression. (A) Venn diagram showing number of DEGs in EAC versus ESCC and LUAD versus LUSC, with overlap. (B) Venn diagram showing number of differentially methylated CpG sites in EAC versus ESCC and LUAD versus LUSC, with overlap. (C) Heatmap depicting relationships in mRNA expression between *TP63* and *TP73*, followed by expression of downstream target genes of p73 transcriptional regulation. (D) Venn diagram showing number of differentially expressed miRNAs in EAC versus ESCC and LUAD versus LUSC, with overlap.
(TIF)

S3 Fig Pathway and upstream regulator analysis of cancer versus normal tissues. (A) Significant pathways from Fig 5, with relationships between four cancer types (EAC, LUAD, ESCC, LUSC) relative to respective normal tissue. (B) Significant upstream regulators from Fig 5, with relationships between four cancer types (EAC, LUAD, ESCC, LUSC) relative to respective normal tissue.
(TIF)

S1 Table Differentially expressed genes corresponding to each functional pathway identified by ingenuity pathway analysis. (XLSX)

S1 File ADC versus SCC differential gene expression data. (XLSX)

S2 File ADC versus SCC differential DNA methylation data. (XLSX)

S3 File ADC versus SCC differential miRNA expression data. (XLSX)
