# Correction: Comparative transcriptomes of adenocarcinomas and squamous cell carcinomas reveal molecular similarities that span classical anatomic boundaries

**DOI:** 10.1371/journal.pgen.1007056

**Published:** 2017-10-18

**Authors:** The PLOS Genetics Staff

Fig 2A is incorrectly duplicated with Fig 1D, due to an error in the production process. Please view the correct version of Fig 2 below. The publisher apologises for the error.

**Fig 2:**
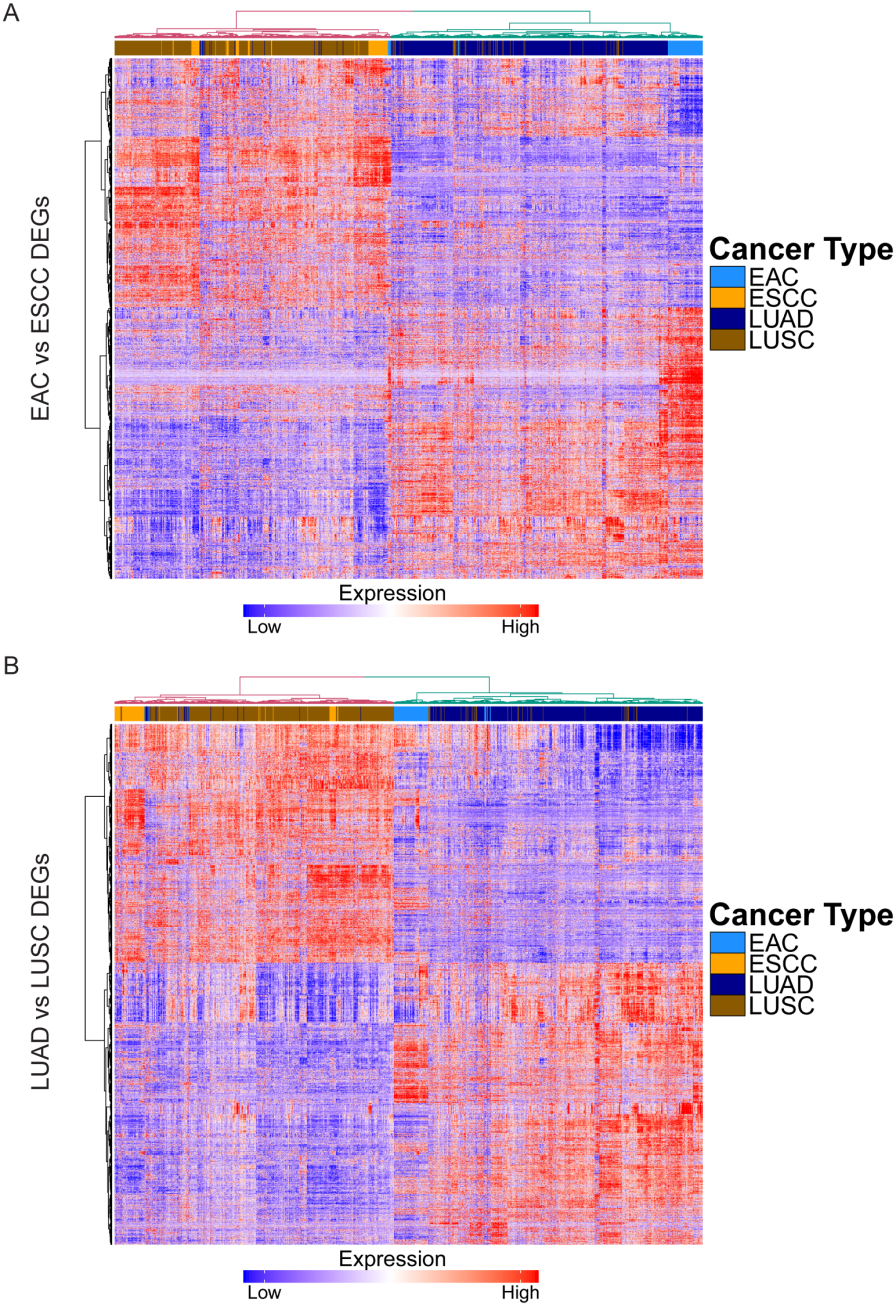
Global molecular patterns defined by histology are consistent across both esophagus and lung. (A) Heatmap depicting mRNA expression of DEGs between EAC and ESCC in ADCs and SCCs of esophagus and lung, with hierarchical clustering. (B) Heatmap depicting mRNA expression of DEGs between LUAD and LUSC in ADCs and SCCs of esophagus and lung, with hierarchical clustering.
