# Supplementary figures and images for: Comparative transcriptomes of adenocarcinomas and squamous cell carcinomas reveal molecular similarities that span classical anatomic boundaries
Source: PLoS Genet. 2017 Aug 7;13(8):e1006938. doi: 10.1371/journal.pgen.1006938 (PMC5560753; doi:10.1371/journal.pgen.1006938)

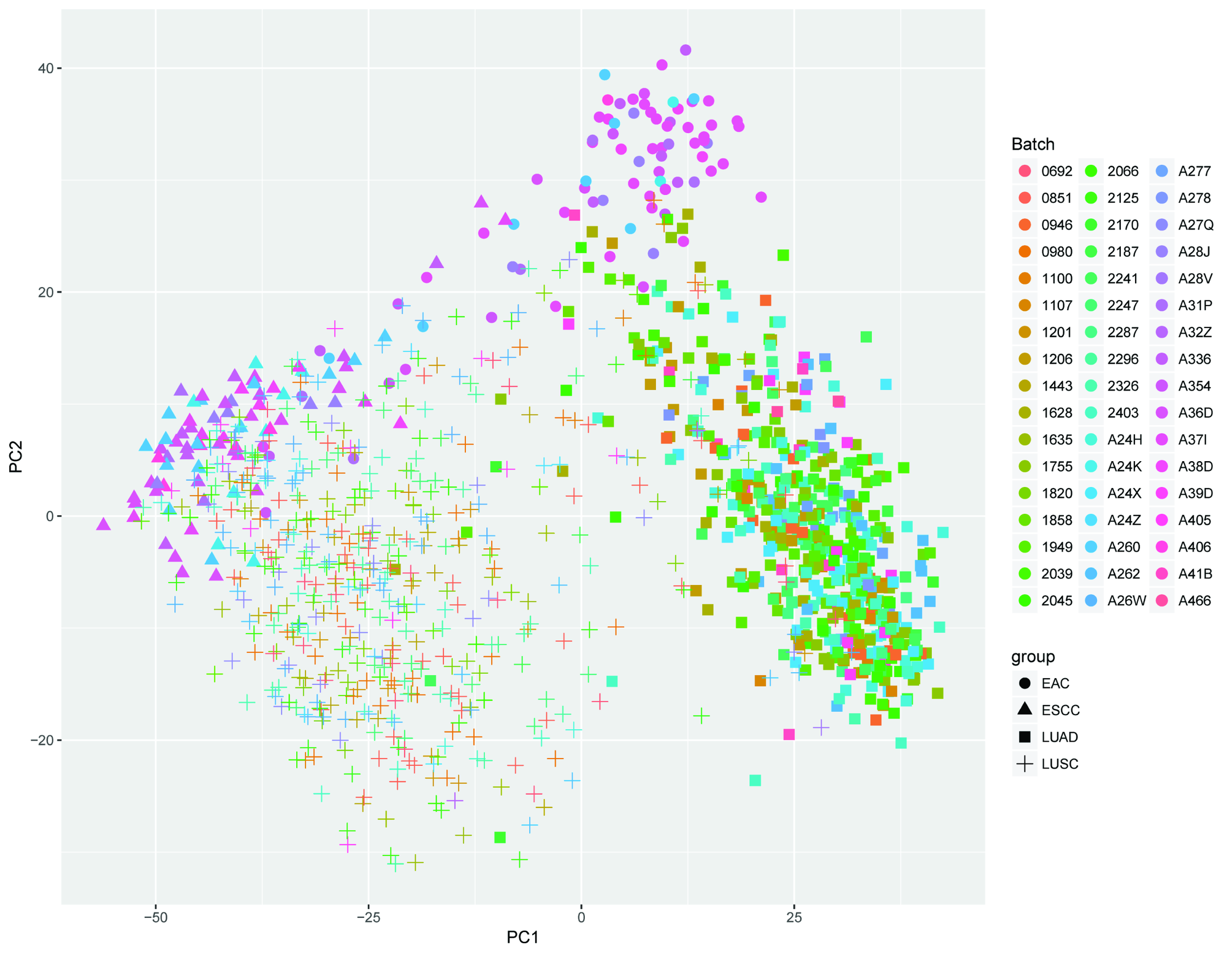

Supplement: S1 Fig — (TIF) [file pgen.1006938.s001.tif]

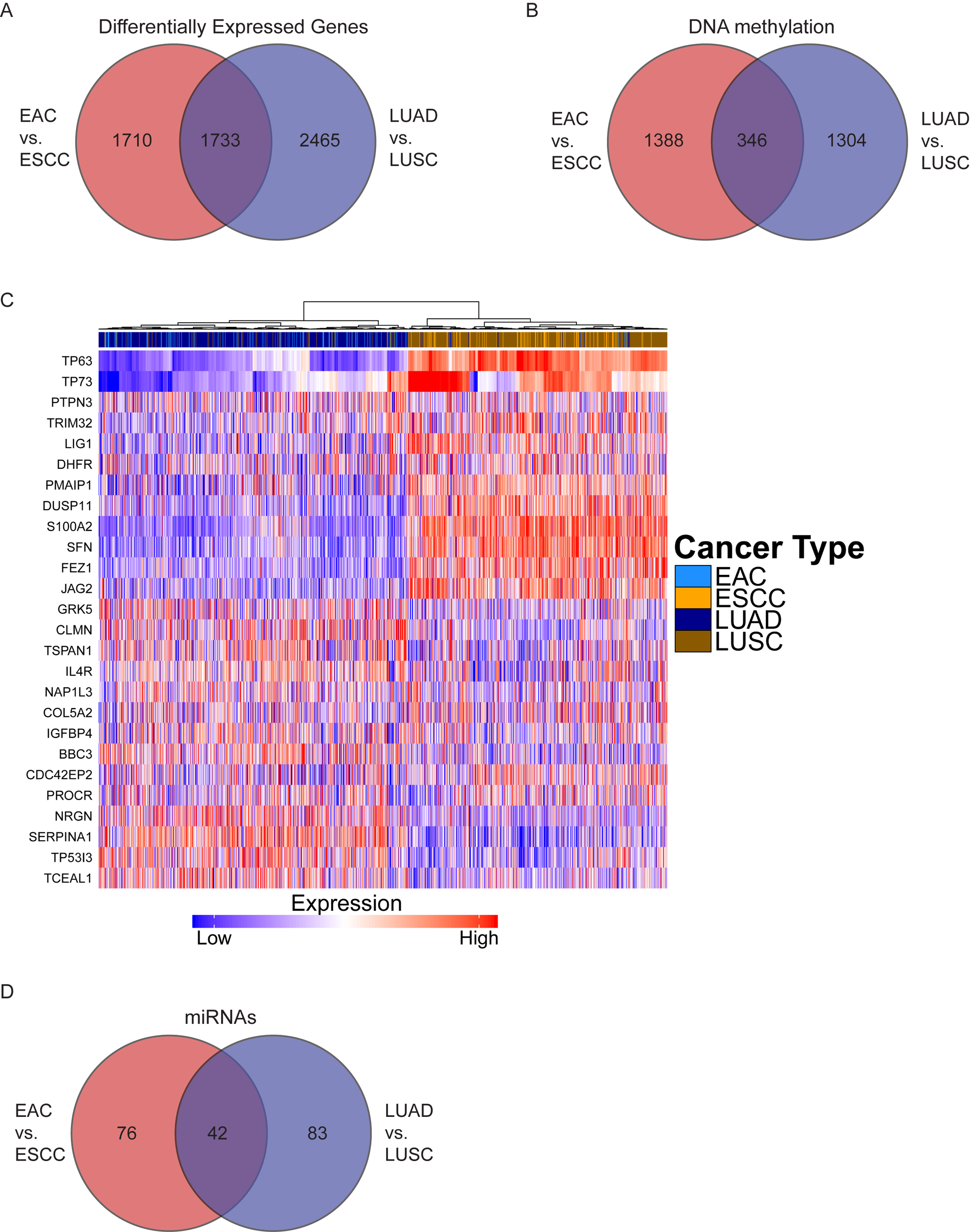

Supplement: S2 Fig — (A) Venn diagram showing number of DEGs in EAC versus ESCC and LUAD versus LUSC, with overlap. (B) Venn diagram showing number of differentially methylated CpG sites in EAC versus ESCC and LUAD versus LUSC, with overlap. (C) Heatmap depicting relationships in mRNA expression between TP63 and TP73, followed by expression of downstream target genes of p73 transcriptional regulation. (D) Venn diagram showing number of differentially expressed miRNAs in EAC versus ESCC and LUAD versus LUSC, with overlap. (TIF) [file pgen.1006938.s002.tif]

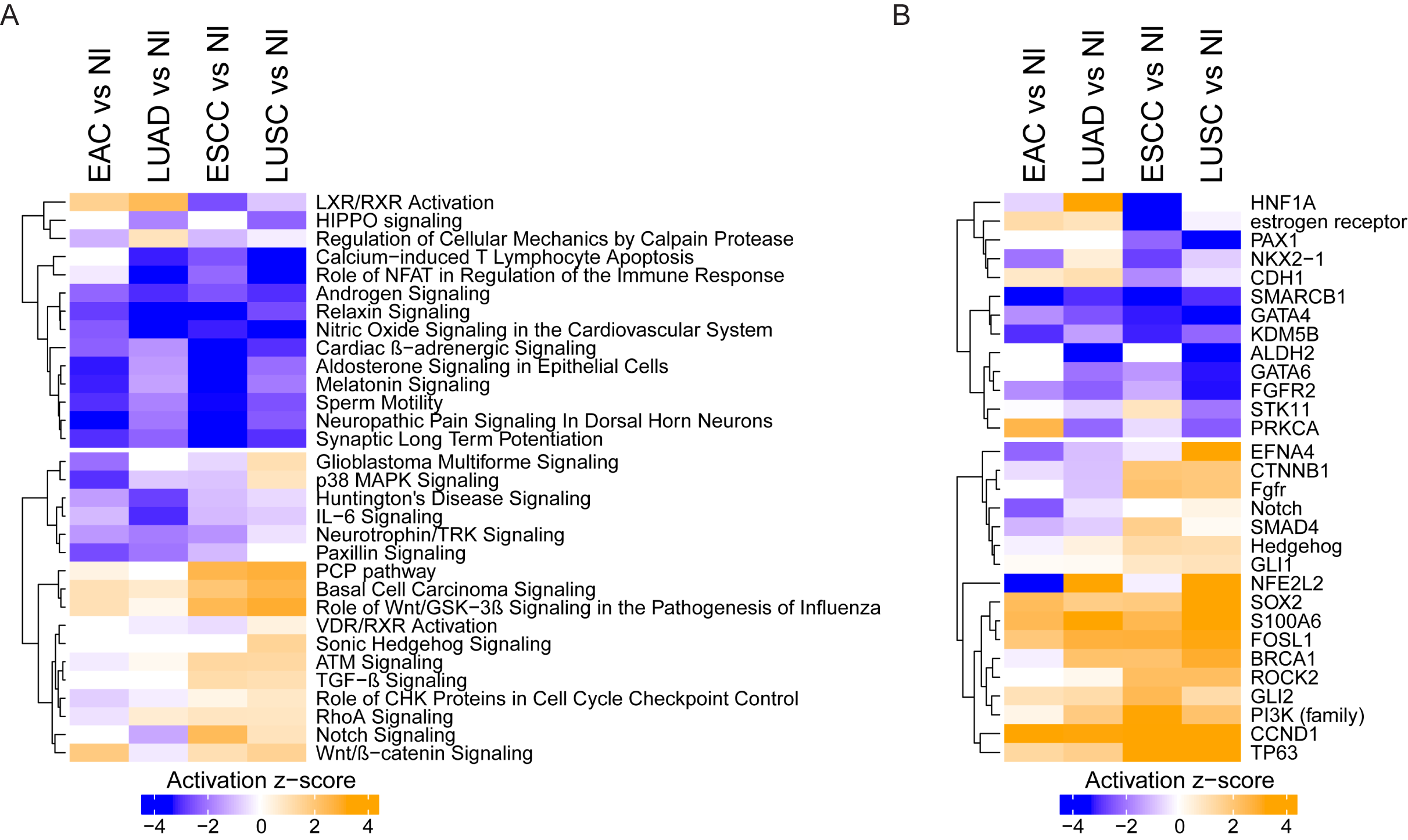

Supplement: S3 Fig — (A) Significant pathways from Fig 5, with relationships between four cancer types (EAC, LUAD, ESCC, LUSC) relative to respective normal tissue. (B) Significant upstream regulators from Fig 5, with relationships between four cancer types (EAC, LUAD, ESCC, LUSC) relative to respective normal tissue. (TIF) [file pgen.1006938.s003.tif]
